# Predictive models of recurrent implantation failure in patients receiving ART treatment based on clinical features and routine laboratory data

**DOI:** 10.1186/s12958-024-01203-z

**Published:** 2024-03-20

**Authors:** Qunying Fang, Zonghui Qiao, Lei Luo, Shun Bai, Min Chen, Xiangjun Zhang, Lu Zong, Xian-hong Tong, Li-min Wu

**Affiliations:** 1 Center for Reproduction and Genetics, Division of Life Sciences and Medicine, The First Affiliated Hospital of USTC, University of Science and Technology of China, Hefei, 230026 Anhui P. R. China; 2 University of Science and Technology of China, Hefei, 230026 Anhui P. R. China

**Keywords:** Recurrent implantation failure, Assisted reproductive technology, Logistic regression analysis, Risk factors

## Abstract

**Study question:**

The objective was to construct a model for predicting the probability of recurrent implantation failure (RIF) after assisted reproductive technology (ART) treatment based on the clinical characteristics and routine laboratory test data of infertile patients.

**Summary answer:**

A model was developed to predict RIF. The model showed high calibration in external validation, helped to identify risk factors for RIF, and improved the efficacy of ART therapy.

**What is known already:**

Research on the influencing factors of RIF has focused mainly on embryonic factors, endometrial receptivity, and immune factors. However, there are many kinds of examinations regarding these aspects, and comprehensive screening is difficult because of the limited time and economic conditions. Therefore, we should try our best to analyse the results of routine infertility screenings to make general predictions regarding the occurrence of RIF.

**Study design, size, duration:**

A retrospective study was conducted with 5212 patients at the Reproductive Center of the First Affiliated Hospital of USTC from January 2018 to June 2022.

**Participants/materials, setting, methods:**

This study included 462 patients in the RIF group and 4750 patients in the control group. The patients’ basic characteristics, clinical treatment data, and laboratory test indices were compared. Logistic regression was used to analyse RIF-related risk factors, and the prediction model was evaluated by receiver operating characteristic (ROC) curves and the corresponding areas under the curve (AUCs). Further analysis of the influencing factors of live births in the first cycle of subsequent assisted reproduction treatment in RIF patients was performed, including the live birth subgroup (*n* = 116) and the no live birth subgroup (*n* = 200).

**Main results and the role of chance:**

(1) An increased duration of infertility (1.978; 95% CI, 1.264–3.097), uterine cavity abnormalities (2.267; 95% CI, 1.185–4.336), low AMH levels (0.504; 95% CI, 0.275–0.922), insulin resistance (3.548; 95% CI, 1.931–6.519), antinuclear antibody (ANA)-positive status (3.249; 95% CI, 1.20-8.797) and anti-β2-glycoprotein I antibody (A-β2-GPI Ab)-positive status (5.515; 95% CI, 1.481–20.536) were associated with an increased risk of RIF. The area under the curve of the logistic regression model was 0.900 (95% CI, 0.870–0.929) for the training cohort and 0.895 (95% CI, 0.865–0.925) for the testing cohort. (2) Advanced age (1.069; 95% CI, 1.015–1.126) was a risk factor associated with no live births after the first cycle of subsequent assisted reproduction treatment in patients with RIF. Blastocyst transfer (0.365; 95% CI = 0.181–0.736) increased the probability of live birth in subsequent cycles in patients with RIF. The area under the curve of the logistic regression model was 0.673 (95% CI, 0.597–0.748).

**Limitations, reasons for caution:**

This was a single-centre regression study, for which the results need to be evaluated and verified by prospective large-scale randomized controlled studies. The small sample size for the analysis of factors influencing pregnancy outcomes in subsequent assisted reproduction cycles for RIF patients resulted in the inclusion of fewer covariates, and future studies with larger samples and the inclusion of more factors are needed for assessment and validation.

**Wider implications of the findings:**

Prediction of embryo implantation prior to transfer will facilitate the clinical management of patients and disease prediction and further improve ART treatment outcomes.

**Study funding/competing interest(s):**

This work was supported by the General Project of the National Natural Science Foundation of China (Nos. 82374212, 81971446, 82301871, and 82201792) and the Natural Science Foundation of Anhui Province (No. 2208085MH206). There are no conflicts of interest to declare.

**Trial registration number:**

This study was registered with the Chinese Clinical Trial Register (Clinical Trial Number: ChiCTR1800018298 ).

**Supplementary Information:**

The online version contains supplementary material available at 10.1186/s12958-024-01203-z.

## Introduction

Infertility, defined as the failure to achieve pregnancy after 12 months of regular unprotected sexual intercourse [1]. According to the World Health Organization, infertility affects approximately 70 million couples of childbearing age worldwide [2]. ART has become an important means to treat infertility. With the continuous development of this technology, the success rate of in vitro fertilization-embryo transfer (IVF-ET) has improved significantly, and implantation rates can reach 60% [3]. However, due to problems such as gamete and embryo quality, endometrial receptivity, and immunity, there are still some patients who do not have successful pregnancies after multiple high-quality embryo transfers; this disease is clinically defined as RIF, which is a challenging clinical problem [4]. The incidence of RIF in IVF-ET patients may be 10–20% [5]. At present, research on the influencing factors of recurrent implantation failure has involved mainly embryonic factors, endometrial receptivity, and immune factors [6–8]. However, there are many kinds of examinations available regarding these aspects, and comprehensive screening is difficult because of the limited time and economic conditions. Therefore, we should try our best to analyse the results of routine infertility screenings of patients to make general predictions regarding the occurrence of RIF. The prediction of embryo implantation prior to transfer facilitates the clinical management of patients and disease prediction. In addition, few studies have reported on the associations between clinical features and laboratory test results and the occurrence of RIF.

This retrospective study was conducted with the help of the clinical database of our centre. Patients undergoing IVF/ICSI cycles from January 2018 to December 2022 were selected from the database to analyse the relationships between the general data and laboratory indicators of infertility patients with RIF, further study the clinical risk factors for RIF, and establish a prediction model for RIF. All patients received symptomatic treatment during IVF if there were abnormal indicators according to the previous routine examination(e.g., HOMA-IR, abnormal hysteroscopy results, ANA, A-β2-GPI Ab, and other immune abnormalities). The influencing factors of pregnancy outcomes in the first cycle of subsequent assisted reproduction treatment in RIF patients were further analysed. This study provides a clinical basis for early treatment and subsequent assisted reproduction treatment in RIF patients to improve IVF/ICSI pregnancy outcomes.

## Materials and methods

### Study population

This study retrospectively analysed infertility patients who received ART treatment at the Reproductive Center of the First Affiliated Hospital of the USTC from January 2018 to June 2022. The patients were divided into a control group (*n* = 4750) and a RIF group (*n* = 462) according to the pregnancy outcome after embryo transfer. The inclusion criteria for the control group were as follows: < 40 years of age and a successful pregnancy after the first IVF/ICSI-ET cycle. The inclusion criteria for the RIF group were as follows: patients who underwent at least 3 cycles of fresh or frozen embryo transfer with a cumulative total of at least 4 good-quality cleavage-stage embryos or 3 blastocysts who still did not achieve a clinical pregnancy; and patients aged < 40 years. The exclusion criteria for patients were as follows: patients for whom embryo transfer was abandoned due to the absence of good-quality embryos for transfer; patients with ovarian hyperstimulation, uterine cavity problems detected on the day of transfer, or other factors; patients with severe anatomical abnormalities of the reproductive tract; patients with a clear history of psychiatric disorders; and patients with contraindications to ART and pregnancy or other comorbid disorders that would have a significant impact on pregnancy.

Data were collected from RIF patients who again received ART treatment between January 2018 and June 2022, and RIF patients who became pregnant after the subsequent first ART cycle were included and categorized into a live birth subgroup (*n* = 116) and a no live birth subgroup (*n* = 200). The aim of this study was to analyse the factors that may affect the pregnancy outcomes of patients with RIF who receive ART treatment after clinical treatment.

**Fig. 1:**
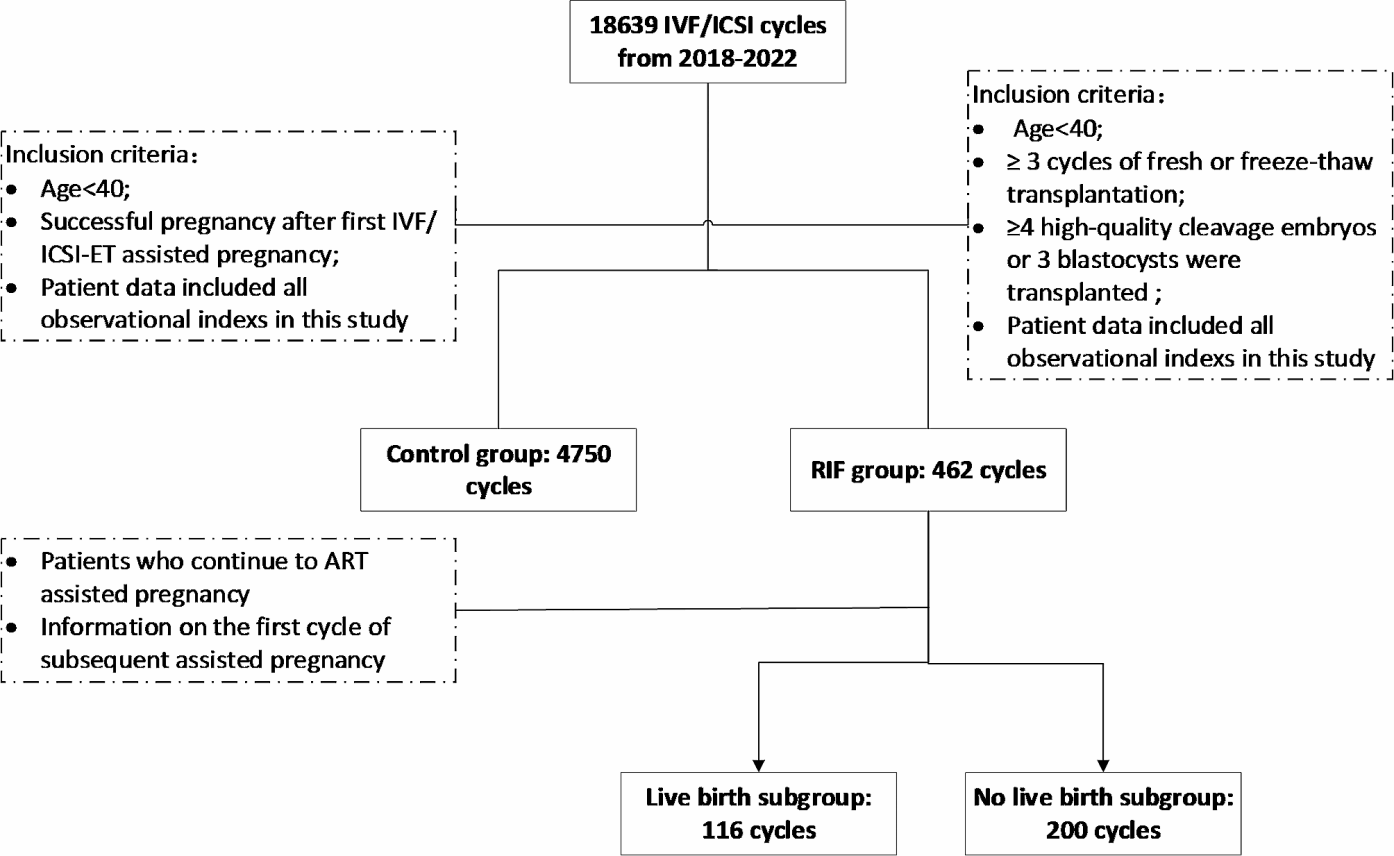
Flow chart of the participant selection process. RIF, recurrent implantation failure; IVF, in vitro fertilization; ICSI, intracytoplasmic sperm injection

This study collected information about the ART protocol used during the enrolment period, including patients’ general data, clinical diagnosis, laboratory indices, and relevant variables during the course of clinical treatment. This study was approved by the Medical Ethics Committee of the First Affiliated Hospital of the USTC. The flow chart of the study population is shown in Fig. 1.

### Covariates

1. The following general clinical data of the patients were collected: age, infertility type, infertility duration, BMI, number of induced abortions, infertility factors, AMH level, basic endocrine hormone level, Homeostatic Model Assessment for Insulin Resistance (HOMA-IR) [HOMA-IR was calculated as follows: fasting insulin (µU/mL)*fasting glucose (mmol/L)/22.5] [9], thyroid function status, karyotype of both couples, hysteroscopy, intrauterine thickness on the day of HCG administration, type of transferred embryo (cleavage or blastocyst), number of transferred embryos, type of infertility, teratozoospermia, and sperm quality [10].
2. The autoimmune antibody-related indices used were as follows: anti-thyroglobulin antibody (TG-Ab), anti-thyroid peroxidase antibody (TPO-Ab), ANA, anticardiolipin antibody (ACA), and A-β2-GPI Ab.

### Statistical analysis

SPSS 23.0 software was used, continuous variable data conforming to a normal distribution are represented by x ± s, and differences between groups were compared by t tests or nonparametric tests. The count data are expressed as n (%), the X2 test was used for univariate categorical variable analysis, and Fisher’s test was used for ordered multicategorical variables. The sample size estimation was calculated by events per variable(EPV), and EPV ≥ 10 [11].

The statistical significance of the univariate analysis was set as two-sided (α = 0.2), and the factors correlated with *P* ≤ 0.2 according to the univariate analysis were selected for multivariate binary logistic regression analysis. A multicollinearity test was performed on the selected predictors. When a variable correlation was found, the variable with the highest correlation with RIF was retained. The step-to-step forward method (likelihood ratio) was adopted, the inclusion standard was 0.05, the exclusion standard was 0.10, and *P* < 0.05 was considered to indicate statistical significance. The odds ratio (OR) and 95% confidence interval (CI) were used to estimate the strength of the association between each factor and the occurrence of RIF. The Box-Tidwell method was used to evaluate the interaction between the continuous independent variable and the natural value in the regression equation. The Hosmer–Lemeshow test was used to determine goodness of fit (*P* < 0.05). We randomly selected 75% of the cycles in the control group and the RIF group to establish a prediction model by using the prediction factors and evaluated the reliability of the RIF prediction model by using the data of the remaining 25% of the cycles. The area under the ROC curve (AUC) was also calculated. The ROC curve represents the sensitivity and 1-specificity of the model, and the AUC value represents the ability of the model to correctly classify the object of study.

## Results

### General clinical data

The general clinical data of the study population are listed in Table 1. There were significant differences between the two groups of patients in terms of age, duration of infertility, proportion of secondary infertility, tubal factor infertility, DOR, EMs, number of induced abortions, age of the male partner and sperm quality (*P* < 0.05).

**Table 1:** General clinical data

	Control (*n* = 4750)	RIF (*n* = 462)	t/X^2^	*P*
Female				
Age (years)	31.00 ± 3.66	33.30 ± 4.14	6.070	0.000
Duration of infertility (years)	2.70 ± 2.04	4.06 ± 2.79	5.721	0.000
BMI (kg/m^2^)	22.92 ± 3.13	22.42 ± 3.02	1.677	0.094
Type of infertility Primary infertility Secondary infertility	2590(54.53) 2160(45.47)	191(41.34) 271(58.66)	29.408	0.000
Diagnosis of infertility				
Tubal factor	3220(67.79)	272(58.87)	15.135	0.000
PCOS	513(10.08)	51(11.04)	0.250	0.875
DOR	290(6.11)	91(19.70)	84.697	0.000
Other ovarian factors	105(2.21)	7(1.52)	0.968	0.325
EMs	430(9.05)	76(16.45)	26.286	0.000
Number of induced abortions			130.388	0.000
0	3315(69.79)	222(48.05)		
1	1050(22.11)	137(29.65)		
≥2	385(8.10)	103(22.30)		
Male				
Age (years)(X ± S)	31.98 ± 4.37	34.56 ± 4.72	5.821	0.000
Type of infertility			13.272	0.000
Primary infertility	2600(54.74)	212(45.89)		
Secondary infertility	2150(45.26)	250(54.11)		
Teratozoospermia	175(3.68)	20(4.33)	0.486	0.486
Sperm quality			192.875	0.000
Normal	2840(59.79)	136(29.44)		
Mild or moderate asthenospermia	1025(21.58)	218(47.19)		
Severe asthenospermia	320(6.74)	49(10.61)		
Others	565(11.89)	59(12.76)		

The data are presented as the mean ± SD or % (n). The data were analysed by ANOVA or the chi-square test and Fisher’s exact test. BMI, body mass index; PCOS, polycystic ovarian syndrome; DOR, diminished ovarian reserve; EMs, endometriosis.

### Laboratory indicators

The laboratory indicators of the study population are listed in Table 2. The results showed that the FSH level, HOMA-IR value, and abnormal proportions of hysteroscopy, TG-Ab, TPO-Ab, ANA, and A-β2-GPI Ab results in the RIF group were significantly greater than those in the control group (*P* < 0.05), and the endometrial thickness on the day of HCG and AMH in the RIF group was significantly lower than that in the control group (*P* < 0.05).

**Table 2:** Laboratory indicators

	Control (*n* = 4750)	RIF (*n* = 462)	t/X^2^	*P*
FSH (IU/L)	7.62 ± 3.35	8.64 ± 3.28	-2.722	0.007
LH (IU/L)	4.84 ± 3.18	4.63 ± 2.18	0.769	0.442
E_2_ (pg/m L)	48.48 ± 20.67	45.59 ± 19.88	1.209	0.228
P (ng/m L)	0.89 ± 2.28	0.81 ± 0.76	0.337	0.736
PRL (ng/m L)	20.89 ± 10.32	20.19 ± 14.61	1.602	0.109
T (ng/m L)	0.43 ± 0.23	0.42 ± 0.25	0.212	0.833
AMH (ng/m L)	4.84 ± 3.71	3.07 ± 2.48	2.585	0.011
FT3 (pmol/L)	4.95 ± 0.6	4.93 ± 0.94	0.138	0.891
FT4 (pmol/L)	17.77 ± 3.56	17.93 ± 3.17	0.166	0.869
TSH (u IU/m L)	2.45 ± 0.3	2.61 ± 0.51	-1.255	0.217
HOMA-IR	1.81 ± 0.519	2.21 ± 0.67	-6.951	0.000
Chromosome karyotype (female partner)			0.332	0.564
Normal abnormal	4570(96.21) 180(3.79)	442(95.67) 20(4.33)		
Chromosome karyotype (male partner)			0.065	0.798
Normal abnormal	4345(91.47) 405(8.53)	421(91.13) 41(8.87)		
Hysteroscopy			184.569	0.000
Normal	3670(77.26)	224(48.48)		
abnormal	1080(22.74)	238(51.52)		
Endometrial thickness on the day of HCG administration (mm)	11.52 ± 1.91	9.54 ± 1.89	5.080	0.000
Immune factor				
ACA			0.362	0.548
Positive Negative	305(6.42) 4445(93.58)	33(7.14) 429(92.86)		
ANA			74.34	0.000
Positive Negative	575(12.11) 4175(87.89)	122(26.41) 340(73.59)		
A-β2-GPI Ab			104.994	0.000
Positive Negative	120(2.53) 4630(97.47)	53(11.47) 409(88.53)		
TG-Ab			73.558	0.000
Positive Negative	495(10.42) 4255(89.58)	110(23.81) 352(76.19)		
TPO-Ab			24.679	0.000
Positive Negative	485(10.21) 4265(89.79)	82(17.75) 380(82.25)		

The data are presented as the mean ± SD or % (n). The data were analysed by ANOVA or the chi-square test and Fisher’s exact test. HOMA-IR was calculated as follows: fasting insulin (µU/mL)*fasting glucose (mmol/L)/22.5. FSH, follicle-stimulating hormone; LH, luteinizing hormone; E2, oestradiol; P, progesterone; PRL, prolactin; T, testosterone; AMH, anti-Müllerian hormone; FT3, free triiodothyronine; FT4, free thyroxin; TSH, thyroid stimulating hormone; HOMA-IR, Homeostatic Model Assessment for Insulin Resistance; ACA, anticardiolipin antibody; ANA, antinuclear antibody; A-β2-GPI, anti-β2-glycoprotein I antibody; TG-Ab, anti-thyroglobulin antibody; TPO-Ab, anti-thyroid peroxidase antibody.

### Correlations of clinical characteristics and laboratory indicators with RIF outcomes

**Fig. 2:**
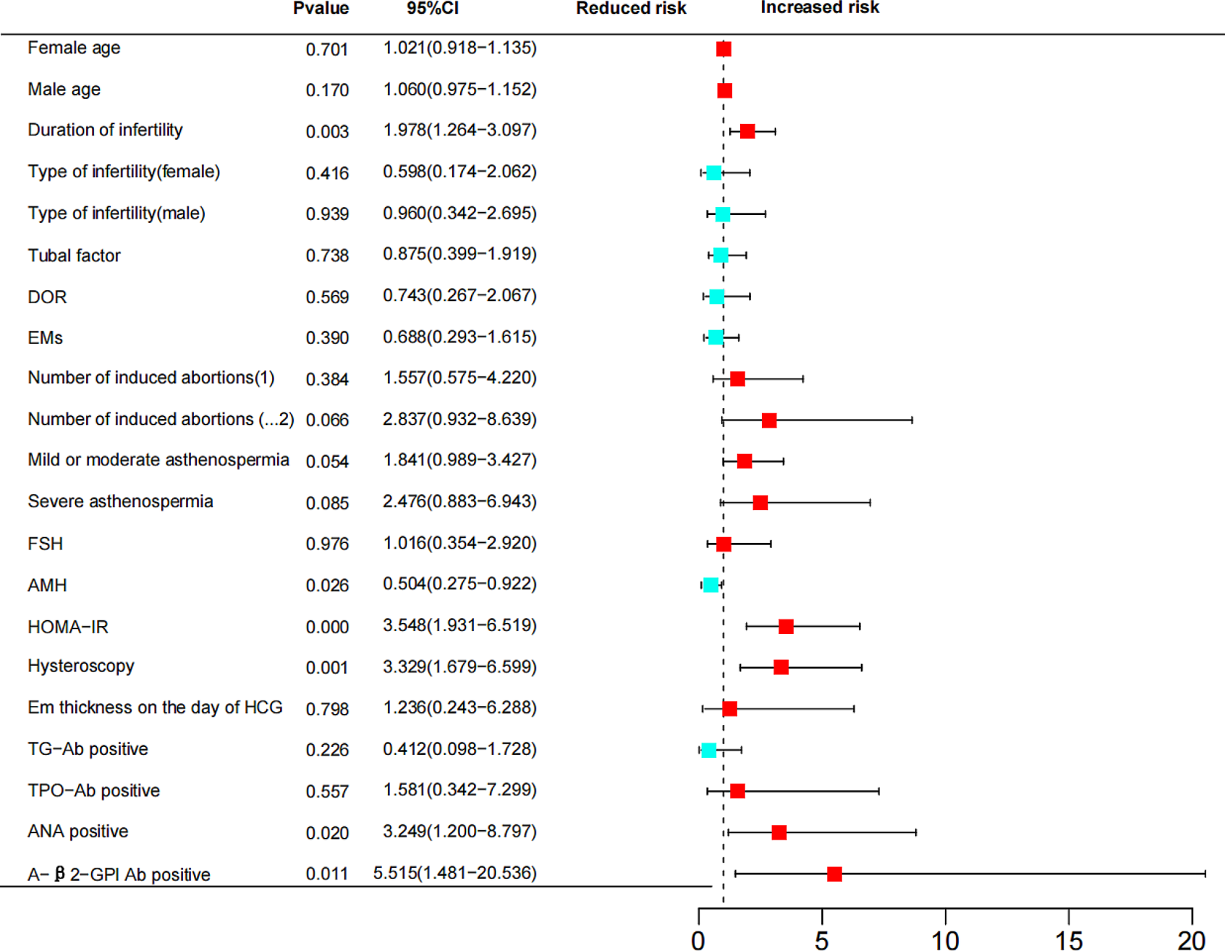
RIF risk forest map of RIF. The duration of infertility, number of induced abortions, low AMH levels, increased HOMA-IR values, abnormal hysteroscopy results, ANA positivity, and A-β2-GPI Ab positivity were risk factors for RIF. DOR, diminished ovarian reserve; EMs, endometriosis; FSH, follicle-stimulating hormone; AMH, anti-Müllerian hormone; HOMA-IR, Homeostatic Model Assessment of Insulin Resistance; TG-Ab, anti-thyroglobulin antibody; TPO-Ab, anti-thyroid peroxidase antibody; ANA, antinuclear antibody; A-β2-GPI Ab, anti-β2-glycoprotein I antibody

**Fig. 3:**
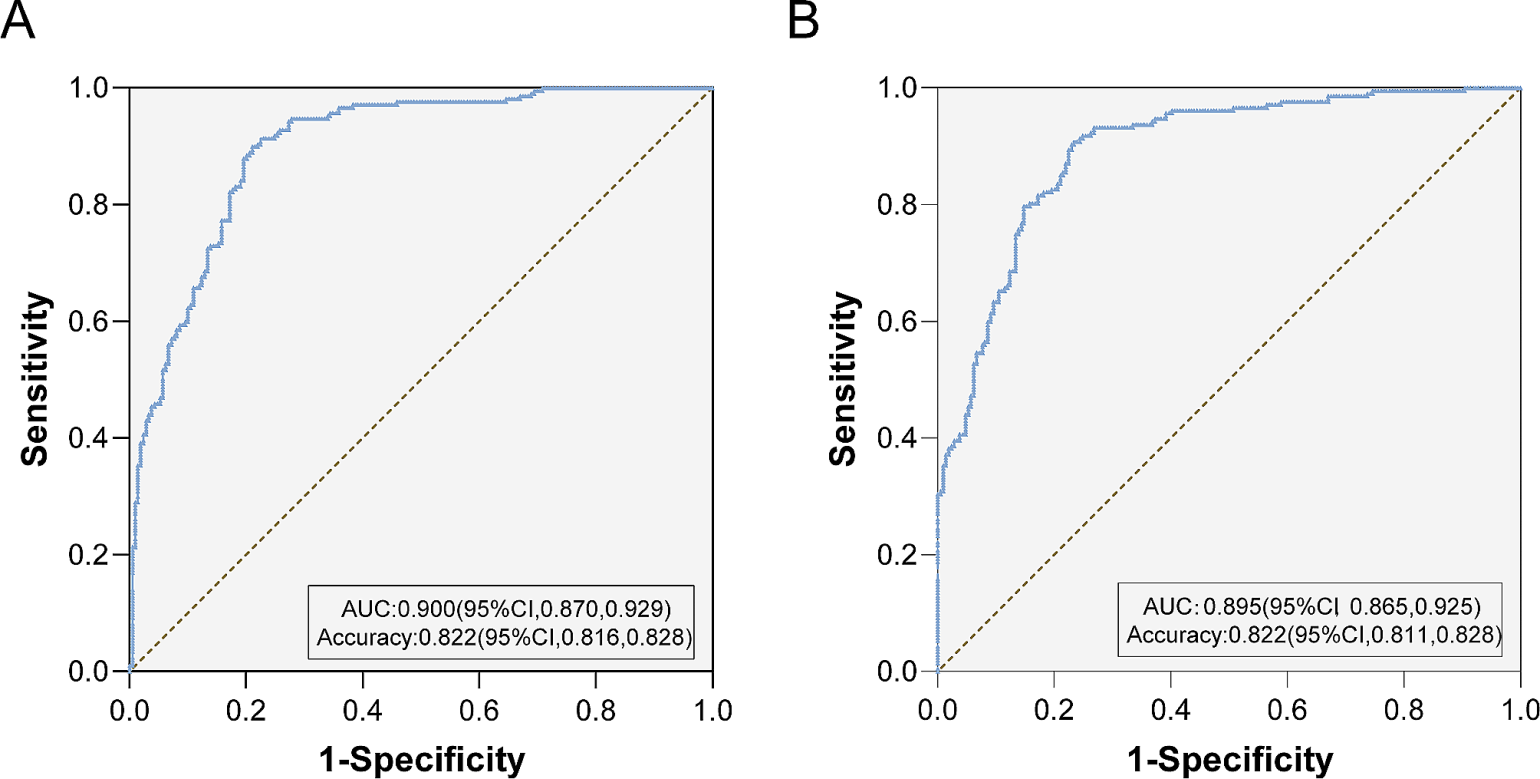
ROC analysis of the training and validation sets. **(A)** The area under the ROC curve (AUC) of the training set was 0.900 (95% CI = 0.870–0.929). **(B)** The AUC of the validation set was 0.895 (95% CI, 0.865∼0.925)

75% of the patients were randomly selected for inclusion in the analysis as a training set for modelling (Table 3 for the values assigned to each predictor). All the included factors were tested for collinearity, which revealed that the variance inflation factor (VIF) was less than 5 and that the tolerances were greater than 0.1, suggesting that there was no multicollinearity among the included factors. The Hosmer and Lemeshow test showed a good model fit (*P* = 0.151). The predictive model showed that longer durations of infertility, abnormal hysteroscopy results, low AMH levels, increased HOMA-IR values, ANA positivity and A-β2-GPI-Ab positivity were associated with an increased risk of RIF (*P* < 0.05). (Table 4**&** Fig. 2.) The ROC curve had an AUC of 0.900 (95% CI, 0.870∼0.929) (*P* = 0.000) (Fig. 3A).

The predictive model was validated against the remaining 25% of patients, and the test cohort ROC curve had an AUC of 0.895 (95% CI, 0.865∼0.925) (*P* = 0.000). Moreover, there was no significant difference between the predictive and test datasets (Fig. 3B).

**Table 3:** Variable assignment

Variables	Assignment
Secondary infertility	Yes (= 1), No (= 0)
Tubal factor	Yes (= 1), No (= 0)
DOR	Yes (= 1), No (= 0)
EMs	Yes (= 1), No (= 0)
Hysteroscopy	Abnormal (= 1), Normal (= 0)
Number of induced abortions	≥ 2 (= 2), 1 (= 1), 0(= 0)
Sperm quality	Severe asthenospermia (= 2), Mild or moderate asthenospermia (= 1), Normal (= 0)
TG-Ab	Positive (= 1), Negative (= 0)
TPO-Ab	Positive (= 1), Negative (= 0)
ANA	Positive (= 1), Negative (= 0)
A-β2-GPI Ab	Positive (= 1), Negative (= 0)

DOR, diminished ovarian reserve; EMs, endometriosis.

### Comparison of the live birth subgroup and the no live birth subgroup after the first cycle of assisted reproduction treatment in RIF patients

After statistical follow-up, 316 RIF patients received ART treatment between January 2018 and June 2022, and 116 patients had live births after the first cycle (live birth subgroup). There were 200 patients without a live birth in the first cycle (no live birth subgroup) (Table 4). The general information and treatment plans of the two groups were compared. The results showed that patient age, endometrial thickness on the day of HCG administration, number of transplanted embryos, and type of transferred embryo were significantly different between the two groups (*P* < 0.05). There were no significant differences in BMI, excretion promotion or endometrial preparation regimens between the two groups (*P* > 0.05) (Table 5).

**Table 4:** Assisted reproduction outcomes of subsequent transfer cycles in RIF patients

Pregnancy outcome	RIF
No. of ET cycles (n)	316
Live birth rate, No. (%)^a^	116/316(36.71)
Biochemical pregnancy rate, No. (%)^b^	149/316(47.15)
Clinical pregnancy rate, No. (%)^c^	135/316(42.72)
Pregnancy loss, No. (%)	33/149(22.15)
Biochemical pregnancy loss	14/149(9.40)
Clinical pregnancy loss	19/149(12.75)

^a^ Live birth was defined as the delivery of a live-born infant after 28 weeks or more of gestation. ^b^ Biochemical pregnancy was defined as a serum human chorionic gonadotropin level greater than 10 mIU/mL. ^c^ Clinical pregnancy was defined as the observation of an intrauterine gestational sac on an ultrasonographic scan.

**Table 5:** General data on the first assisted reproduction cycle in patients with RIF

	Live birth subgroup (n = 116)	No live birth subgroup (n = 200)	t/X^2^	*P* value
Age (years)	33.09 ± 5.14	35.41 ± 6.16	-2.930	0.004
BMI (kg/m^2^)	22.63 ± 2.90	22.89 ± 2.64	-0.654	0.514
Ovarian hyperstimulation/Endometrial preparation protocols	33	120	1.086	0.909
Fresh cycle	14	55		
Long protocol	2	6		
Super long protocol	6	20		
Modified super long protocol	6	24		
Antagonist protocol	6	15	0.445	0.8
Microstimulation protocol Frozen-thawed cycle	83 7	80 8		
Natural cycle Hormone therapy cycle	60 16	54 18		
Ovulation induction cycle				
Endometrial thickness on the day of HCG administration (mm)	10.02 ± 1.68	9.43 ± 2.23	2.153	0.033
No. of embryos transferred			4.541	0.039
1 2	51 65	64 136		
Type of embryos transferred			13.181	0.000
Cleavage embryo	62	147		
Blastocyst	54	53		

The data are presented as the mean ± SD or % (n). The data were analysed by ANOVA or the chi-square test and Fisher’s exact test. BMI, body mass index.

### Multivariate analysis of pregnancy assistance in the first cycle of RIF

**Fig. 4:**
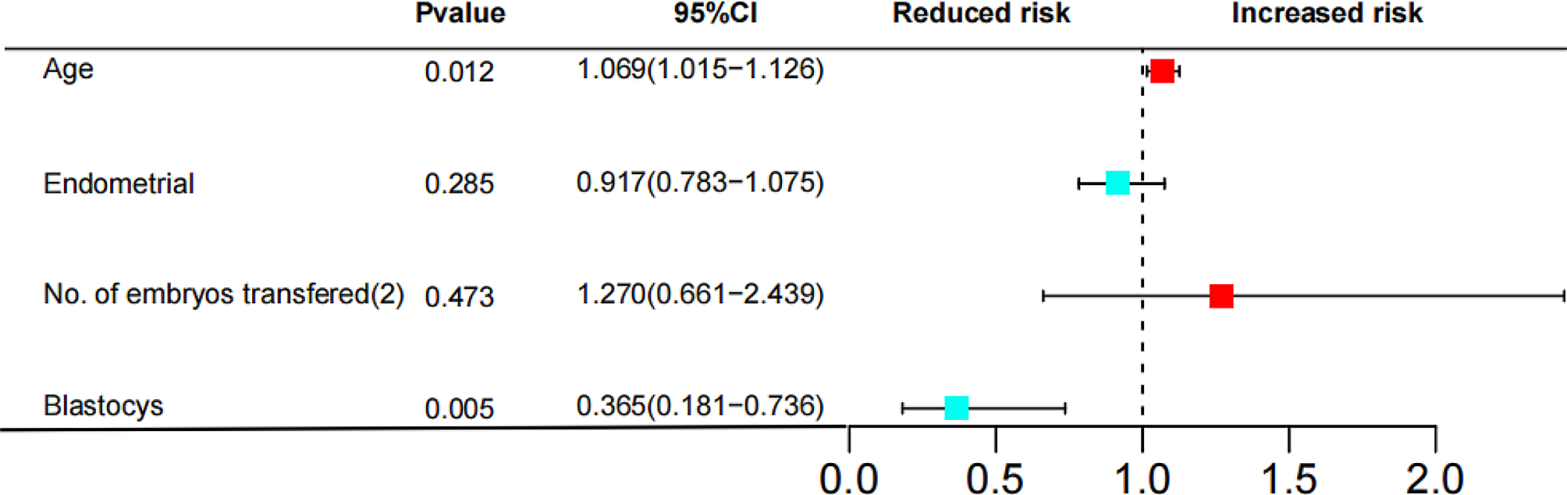
RIF risk forest map Age was a risk factor for no live births in patients with RIF (*P* = 0.012, 95% CI = 1.069 (1.015 ~ 1.126)). Blastocyst transfer was a protective factor against unsuccessful pregnancy in RIF patients (*P* = 0.005, 95% CI = 0.365 [0.181 ~ 0.736])

Logistic regression analysis (same method as above) was used to analyse the different influencing factors in the univariate analysis. The results showed that the age of the patient and the type of embryo transferred were risk factors for no live births in the first assisted reproduction cycle after RIF (*P* < 0.05) (Fig. 4). Logistic regression analysis revealed no collinearity among the included factors, and there was no significant difference between the Hosmer and Lemeshow test results (*P* = 0.733), indicating that the model was a good fit. The logistic regression analysis prediction model was evaluated by the ROC curve (AUC = 0.673) (95% CI, 0.597∼0.748) (*P* = 0.000) (**Fig. **S1).

## Discussion

In this study, the objective was to construct a model for predicting the probability of recurrent implantation failure (RIF) after assisted reproductive technology (ART) treatment based on the clinical characteristics and routine laboratory test data of infertile patients. The results revealed that increased infertility duration, uterine cavity abnormalities, low AMH levels, insulin resistance, ANA positivity, and A-β2-GPI Ab positivity were associated with an increased risk of RIF. In subsequent ART cycles for RIF patients, advanced age increases the risk of no live births, and blastocyst transplantation is more conducive to achieving a live birth.

With the development of ART, clinical treatment protocols are becoming increasingly effective, and the success rate of IVF has improved; however, there are still some families who undergo multiple high-quality embryo transfers for fertility treatment and are unable to achieve pregnancy. With the development of preimplantation genetic testing (PGT), approximately 70% of embryos are identified as high quality [12]. Thus, maternal autopathological factors are closely related to the occurrence of RIF. In previous studies, aetiological screening for RIF included examination of thrombosis tendency, immunoglobulin levels, lymphocyte subsets, and multiple immune cytokine profiles [13–15]. However, the significance of several indicators in the diagnosis and prediction of RIF is still controversial. Patients with RIF suffer great psychological and economic pressure due to multiple implantation failures. Therefore, analysing the clinical characteristics of patients and the results of routine laboratory tests are particularly important for the analysis and prediction of RIF to provide patients with more reasonable clinical management strategies to improve pregnancy outcomes.

With increasing infertility duration, sperm may exhibit decreased acrosomal protein activity and nuclear chromatin immaturity, which increases the possibility of sperm–egg union disorder in infertile couples [16]. This increases the risk of embryo implantation failure. The results of this study suggest that an increase in infertility duration is a risk factor for RIF. Studies have shown that pregnancy rates are closely related to a woman’s age [17]. The proportion of oocytes with chromosomal abnormalities begins to increase after the age of 26 years, and the older the woman is, the greater the probability of chromosomal abnormalities in oocytes [18], which may affect embryonic development potential. Previous studies have shown that primary infertility is associated with IVF-assisted pregnancy failure [19]. In this study, there were significant differences in age and infertility type between the RIF group and the control group, but logistic regression analysis showed that age and infertility type were not risk factors for RIF. In future studies, data from more centres and larger samples are needed for verification.

Embryo implantation requires a good uterine environment, and studies have shown that the incidence of uterine cavity abnormalities in RIF patients can reach 25–50% [20]. Ultrasonography and hysteroscopy are commonly used in clinical monitoring. The most common intrauterine lesions closely related to embryo implantation include intrauterine adhesions, endometritis, endometrial polyps and submucosal myoma. Hysteroscopy is the gold standard for detecting and treating uterine factors and can detect intrauterine lesions that may be missed by other examinations [21]. Most studies suggest that patients with RIF should undergo hysteroscopy before undergoing further assisted reproduction cycles and that pregnancy should be facilitated after ruling out/treating uterine cavity lesions, therefore significantly improving the pregnancy rate [22]. In this study, the proportion of uterine abnormalities in patients with RIF was significantly greater than that in healthy individuals, and logistic regression analysis revealed that uterine abnormalities were a risk factor for RIF.

AMH is a member of the transforming growth factor β (TGF-β) superfamily and is secreted by ovarian granulosa cells [23]. The serum AMH concentration is widely used to evaluate and predict ovarian function, COH, embryo quality and pregnancy outcomes [24, 25]. Previous studies have shown that the AMH concentration is an important clinical predictor of ART cycle outcomes [26]. In a study based on single-dominant follicles and in vitro fertilization (IVF), AMH levels in follicular fluid, but not in serum, were correlated with embryo implantation potential [27]. In this study, the AMH concentration in the RIF group was significantly lower than that in the control group, and logistic regression analysis revealed that a low AMH concentration was a risk factor for the occurrence of RIF. Low level of AMH may affect the quality of embryos and thus the implantation of embryos.

Studies have shown that IR can affect oocyte meiosis in PCOS patients and delay oocyte maturation, thereby reducing the number of mature oocytes [28]. A study in a mouse model of insulin resistance showed that IR increased oxidative stress and mitochondrial dysfunction in mouse oocytes, resulting in poor oocyte quality and reduced fertility [29]. A prospective clinical study showed that the implantation and pregnancy rates of PCOS patients with IR were significantly lower than those of PCOS patients without IR, and there was no significant difference in embryo quality between the two groups. It is speculated that IR may reduce the embryo implantation rate by affecting the function of the endometrium in patients [30]. In this study, the HOMA-IR score was significantly greater in RIF patients than in control individuals. Logistic regression analysis revealed that a high IR was a risk factor for the occurrence of RIF.

Previous studies have shown that autoimmune coordination is an important condition for a successful pregnancy [31, 32]. ANA is a screening antibody for autoimmune diseases. ANA positivity can reduce oocyte quality, affect embryo development, and reduce the embryo implantation and pregnancy rates, resulting in repeated pregnancy loss [33–35]. Anti-phospholipid antibodies (APAs), such as anti-cardiolipin (aCL) and A-β2-GPI Abs, can affect pregnancy outcomes by interfering with oocyte development, embryo morphology, uterine contractions, and appropriate decidua and are potential causes of hypofertility [36]. In this study, the percentages of patients who tested positive for thyroid antibodies, ANAs and A-β2-GPI Abs in the RIF group were significantly greater than those in the control group. Logistic regression analysis revealed that ANA and A-β2-GPI Ab positivity were risk factors for the occurrence of RIF.

Aetiological screening was carried out for RIF patients, and assisted reproduction therapy was continued after symptomatic treatment. This study further analysed RIF patients with and without live births after the subsequent first assisted reproduction cycle. Logistic regression analysis revealed that advanced age was a risk factor for no live births after subsequent assisted reproduction cycles in RIF patients, and blastocyst transfer improved the live birth rate after subsequent assisted reproduction cycles in RIF patients. Advanced age leads to a decrease in the number of transferable embryos and strongly affects the quality of embryos; in particular, an increase in the number of aneuploid embryos significantly reduces the pregnancy rate [37, 38]. It has been reported that with increasing age, the asynchronism of embryo-intima development increases. In women aged 35 years, the increase in asynchronism of the embryo intima can lead to a significant decrease in the embryo implantation rate and a significant increase in the biochemical pregnancy rate [39]. Therefore, for RIF patients undergoing subsequent assisted reproduction cycles, the appropriate length of time should be determined to improve the live birth rate. A prospective cohort study showed that implantation rates were significantly greater in patients who underwent blastocyst transfer than in patients who underwent cleavage-stage embryo transfer [40]. Studies have shown that blastocyst transfer can improve the clinical pregnancy rate in patients with RIF [41]. Therefore, for the subsequent assisted pregnancy cycle of RIF patients, blastocyst transfer should be performed as much as possible, and assisted reproduction treatment should be continued as soon as possible through reasonable cycle management to minimize the adverse effects of age on assisted pregnancy outcomes.

### Limitations

This was a single-centre regression study, for which the results need to be evaluated and verified by prospective large-scale randomized controlled studies. The small sample size for the analysis of factors influencing pregnancy outcomes in subsequent assisted reproduction cycles for RIF patients resulted in the inclusion of fewer covariates, and future studies with larger samples and the inclusion of more factors are needed for assessment and validation.

## Conclusion

In summary, an increased infertility duration, uterine cavity abnormalities, low AMH levels, insulin resistance, ANA positivity, and A-β2-GPI Ab positivity were associated with an increased risk of RIF. In subsequent ART cycles for RIF patients, advanced age increases the risk of no live births, and blastocyst transplantation is more conducive to achieving a live birth.

## Electronic supplementary material

Below is the link to the electronic supplementary material.

Supplementary Material 1

Supplementary Material 2. Figure S1. Prediction model of subsequent first ET cycles in RIF patients The area under the ROC curve (AUC) of the training set was 0.673 (95% CI: 0.597∼0.748).

## Data Availability

The data underlying this article will be shared upon reasonable request to the corresponding author.
