# Correction: Predictive models of recurrent implantation failure in patients receiving ART treatment based on clinical features and routine laboratory data

**DOI:** 10.1186/s12958-024-01218-6

**Published:** 2024-04-17

**Authors:** Qunying Fang, Zonghui Qiao, Lei Luo, Shun Bai, Min Chen, Xiangjun Zhang, Lu Zong, Xian-hong Tong, Li-min Wu

**Affiliations:** 1 Center for Reproduction and Genetics, Division of Life Sciences and Medicine, The First Affiliated Hospital of USTC, University of Science and Technology of China, Hefei, 230026 Anhui P. R. China; 2 University of Science and Technology of China, Hefei, 230026 Anhui P. R. China

**Correction: Reprod Biol Endocrinol 22, 32 (2024)**

10.1186/s12958-024-01203-z

Following publication of the original article [1], the authors reported an alignment error in Table 1 and typos found under section headings Correlations of clinical characteristics and laboratory indicators with RIF outcomes; Multivariate analysis of pregnancy assistance in the first cycle of RIF; Fig. 3 caption; Supplementary Material 1 caption and the author’s unit.

**Table 1:** General clinical data (Corrected)

	Control	RIF	t/X^2^	*P*
	(n = 4750)	(n = 462)		
Female				
Age (years)	31.00 ± 3.66	33.30 ± 4.14	6.07	0
Duration of infertility (years)	2.70 ± 2.04	4.06 ± 2.79	5.721	0
BMI (kg/m^2^)	22.92 ± 3.13	22.42 ± 3.02	1.677	0.094
Type of infertility			29.408	0
Primary infertility	2590(54.53)	191(41.34)		
Secondary infertility	2160(45.47)	271(58.66)		
Diagnosis of infertility				
Tubal factor	3220(67.79)	272(58.87)	15.135	0
PCOS	513(10.08)	51(11.04)	0.25	0.875
DOR	290(6.11)	91(19.70)	84.697	0
Other ovarian factors	105(2.21)	7(1.52)	0.968	0.325
Ems	430(9.05)	76(16.45)	26.286	0
Number of induced abortions			130.388	0
0	3315(69.79)	222(48.05)		
1	1050(22.11)	137(29.65)		
≥2	385(8.10)	103(22.30)		
Male				
Age (years)(X ± S)	31.98 ± 4.37	34.56 ± 4.72	5.821	0
Type of infertility			13.272	0
Primary infertility	2600(54.74)	212(45.89)		
Secondary infertility	2150(45.26)	250(54.11)		
Teratozoospermia	175(3.68)	20(4.33)	0.486	0.486
Sperm quality			192.875	0
Normal	2840(59.79)	136(29.44)		
Mild or moderate asthenospermia	1025(21.58)	218(47.19)		
Severe asthenospermia	320(6.74)	49(10.61)		
Others	565(11.89)	59(12.76)		

The data are presented as the mean ± SD or % (n). The data were analysed by ANOVA or the chi-square test and Fisher’s exact test

BMI, body mass index; PCOS, polycystic ovarian syndrome; DOR, diminished ovarian reserve; EMs, endometriosis

The heading Correlations of clinical characteristics and laboratory indicators with RIF outcomes, in this part of the results “95% CI, 0.870 to 0.929” was modified to“95% CI, 0.870 ∼ 0.929”.

The heading Correlations of clinical characteristics and laboratory indicators with RIF outcomes, in this part of the results, “95% CI = 0.865➔0.925” modified to“95% CI, 0.865 ∼ 0.925”.

The heading Multivariate analysis of pregnancy assistance in the first cycle of RIF, in this part of the results, “95% CI, 0.597➔0.748” was modified to “95% CI, 0.597 ∼ 0.748”.

In Fig. 3 caption, “0.895 (95% CI = 0.811–0.828)” was modified to“95% CI, 0.865 ∼ 0.925”.

In Supplementary Material 1 caption, “95% CI: 0.597?0.748” was modified to “95% CI, 0.597 ∼ 0.748”.

In author details, “Center for reproductive medicine and prenatal diagnosis ” was modified to “Center for Reproduction and Genetics”.

The original article [1] has been updated.
