# Correction: Predictive models of recurrent implantation failure in patients receiving ART treatment based on clinical features and routine laboratory data

**DOI:** 10.1186/s12958-024-01308-5

**Published:** 2024-11-05

**Authors:** Qunying Fang, Zonghui Qiao, Lei Luo, Shun Bai, Min Chen, Xiangjun Zhang, Lu Zong, Xian-hong Tong, Li-min Wu

**Affiliations:** 1 Center for Reproduction and Genetics, Division of Life Sciences and Medicine, The First Affiliated Hospital of USTC, University of Science and Technology of China, Hefei, 230026 Anhui P. R. China; 2 University of Science and Technology of China, Hefei, 230026 Anhui P. R. China

**Correction: Reprod Biol Endocrinol 22, 32 (2024)**

10.1186/s12958-024-01203-z

Following publication of the original article [1], the authors reported an error found in the Funding section.

Current funding statement: This work was supported by the General Project of the National Natural Science Foundation of China (Nos. **82201792**,** 82301871**,** 81971446**,** and 82374212**) and the Natural Science Foundation of Anhui Province (No. 2208085MH206).

Funding statement updated to: This work was supported by the General Project of the National Natural Science Foundation of China (Nos. **82374212**,** 81971446**,** 82301871 and 82201792**) and the Natural Science Foundation of Anhui Province (No. 2208085MH206).

The original article [1] has been updated.

## References

[1] Fang Q, Qiao Z, Luo L, et al. Predictive models of recurrent implantation failure in patients receiving ART treatment based on clinical features and routine laboratory data. Reprod Biol Endocrinol. 2024;22:32. 10.1186/s12958-024-01203-z. 38509534 10.1186/s12958-024-01203-z PMC10953148

