# Supplementary material for: Predictive models of recurrent implantation failure in patients receiving ART treatment based on clinical features and routine laboratory data
Source: Reprod Biol Endocrinol. 2024 Mar 20;22:32. doi: 10.1186/s12958-024-01203-z (PMC10953148; doi:10.1186/s12958-024-01203-z)
Supplement: Supplementary file 1 — Supplementary Material 1 [file 12958_2024_1203_MOESM1_ESM.pdf]

SPRINGER NATURE

Author Services

Editing Certificate

This document certifies that the manuscript

Predictive models of recurrent implantation failure in patients receiving ART treatment based on clinical features and routine laboratory data

prepared by the authors

Qunying Fang, Zonghui Qiao, Lei Luo, Shun Bai, Min Chen, Xiangjun Zhang, Lu Zong1, Xian-hong Tong, Li-min Wu

was edited for proper English language, grammar, punctuation, spelling, and overall style by one or more of the highly qualified native English speaking editors at SNAS.

This certificate was issued on **January 22, 2024** and may be verified on the SNAS website using the verification code **8A73-7BA4-68C8-1736-7F8A**.

Neither the research content nor the authors' intentions were altered in any way during the editing process. Documents receiving this certification should be English-ready for publication; however, the author has the ability to accept or reject our suggestions and changes. To verify the final SNAS edited version, please visit our verification page at secure.authorservices.springernature.com/certificate/verify.
If you have any questions or concerns about this edited document, please contact SNAS at support@as.springernature.com.

SNAS provides a range of editing, translation, and manuscript services for researchers and publishers around the world.
For more information about our company, services, and partner discounts, please visit authorservices.springernature.com.
