# Supplementary material for: Predictive models of recurrent implantation failure in patients receiving ART treatment based on clinical features and routine laboratory data
Source: Reprod Biol Endocrinol. 2024 Mar 20;22:32. doi: 10.1186/s12958-024-01203-z (PMC10953148; doi:10.1186/s12958-024-01203-z)
Supplement: Supplementary file 2 — Supplementary Material 2. Figure S1. Prediction model of subsequent first ET cycles in RIF patients The area under the ROC curve (AUC) of the training set was 0.673 (95% CI: 0.597∼0.748). [file 12958_2024_1203_MOESM2_ESM.docx]

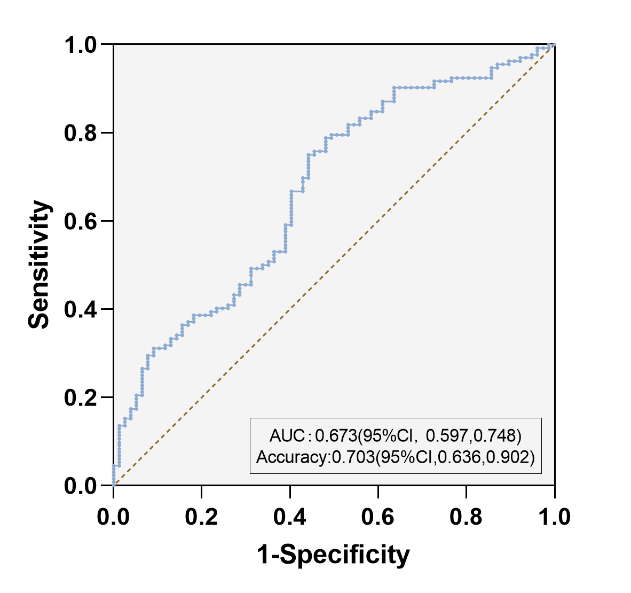


**Figure S1. Prediction model for subsequent ET cycles in RIF patients** The area under the ROC curve (AUC) of the training set was 0.673 (95% CI=0.597–0.748).
